# Comparative secretomic and *N*-glycoproteomic profiling in human MCF-7 breast cancer and HMEpC normal epithelial cell lines using a gel-based strategy

**DOI:** 10.1186/s12935-014-0120-x

**Published:** 2014-11-30

**Authors:** Aik-Aun Tan, Alan Kang-Wai Mu, Lik-Voon Kiew, Yeng Chen

**Affiliations:** Institute for Research in Molecular Medicine (INFORMM), Universiti Sains Malaysia, Penang, 11800 Malaysia; Department of Oral Biology and Biomedical Sciences, Faculty of Dentistry, University of Malaya, Kuala Lumpur, 50603 Malaysia; Oral Cancer Research and Coordinating Centre, Faculty of Dentistry, University of Malaya, Kuala Lumpur, 50603 Malaysia; Department of Pharmacology, Faculty of Medicine Building, University of Malaya, Kuala Lumpur, 50603 Malaysia

**Keywords:** Secretomic, Glycoproteomic, Two dimensional electrophoresis, *N*-glycoprotein, Concanavalin A lectin

## Abstract

**Background:**

Concurrent study of secretomic and glycoproteomic profiles in cancer cell lines represents an excellent approach for investigating cancer progression and identifying novel biomarker candidates. In this study, we performed a comparative secretomic and *N-*glycoprotein profiling from the secretions of normal human mammary epithelial cells (HMEpC) and the MCF-7 human breast cancer cell line.

**Method:**

We analyzed these cell lines using a combined methodology involving glycan-binding lectins and two-dimensional electrophoresis and identified several differentially secreted factors, including osteonectin and haptoglobin.

**Result:**

Notably, comparative analyses also revealed that MCF-7 cells produced differentially *N*-glycosylated forms of haptoglobin.

**Conclusion:**

The present data suggested that osteonectin and haptoglobin might have potential to be served as potential biomarkers for breast cancer. However, further investigation is needed to validate the findings.

## Introduction

Breast cancer is one of the most common cancers affecting women worldwide, accounting for almost 23% of all cancers diagnosed in women [1,2]. Although the mortality rate has decreased by 26.58% from 1991 to 2005, breast cancer remains as one of the leading causes of death in women [3]. This is mainly due to late stage diagnosis and lack of effective treatments. Therefore, early detection is critical for successful therapy and ultimate survival of breast cancer patients.

Human breast cancer cell lines provide an excellent tool for investigating tumor progression and therapy. In fact, secretome profiling of cell lines could represent an efficient method for discovering novel biomarkers for early breast cancer detection [4]. According to Lai et al., false positive results obtained from tumor specimens could be minimized through the identification of potential diagnostic proteins that are secreted by cancer cells [5]. During tumorigenesis, it is known that secreted proteins can directly participate in cancer cell migration, invasion, growth control, angiogenesis, matrix degradation, and adhesion [6]. Therefore, analysis of these tumor-secreted factors not only contributes to our knowledge of the molecular mechanisms involved in tumorigenesis and metastasis, but also represents a promising strategy for identifying biomarkers.

Glycosylation is an enzymatic process that attaches glycans to proteins, lipids, or other organic molecules. Glycosylation functionally contributes to protein stability as well as cell–cell recognition and communication [7]. For this reason, modifications in glycans can contribute to tumorigenesis. Changes in glycans include lost or excessive expression of certain structures, incomplete/truncated structures, accumulation of precursors, and/or altered glycan expression [8]. Indeed, modified glycosylation patterns are associated with cancer invasiveness and metastatic potential [9]. Most of the secreted proteins are known to be *N*-glycosylated [10]. Therefore, comparative studies of *N*-glycosylation profiles of secreted proteins in malignant cell lines and normal cell lines could provide important clues related to tumor diagnosis and prognosis.

In this study, we conducted a comparative analysis of *N-*glycoprotein secretion from normal human mammary epithelial cells (HMEpC) and the MCF-7 human breast cancer cell line, which is of luminal epithelial origin and used as a model for estrogen receptor-positive tumors. To detect *N*-glycoproteins secreted from these cell lines we utilized Concanavalin A (ConA) extracted from *Concanavalia ensiformis*, which selectively recognizes alpha-mannose of *N*-glycan. Herein, we have coupled the use of these glycan-binding lectins with two-dimensional electrophoresis (2-DE) in order to identify proteins that are aberrantly secreted and/or glycosylated in MCF-7 cells.

## Materials and method

### Cell culture

The human breast cancer cell line, MCF-7 (catalog number: HTB-22), and human mammary epithelial cell line, HMEpC (catalog number: 830 K-05a), were purchased from American Type Culture Collection (ATCC) and Cell Applications, respectively.

### Sampling of growth medium

Similar amount of MCF-7 and HMEpC cells line were cultured in separate flasks with FBS-containing growth media and human mammary epithelial cell growth medium, respectively. The growth medias were removed when cells were approximately 80% confluent (10^8^ cells) in a 75 cm^2^ flask. The cells were then washed three times with phosphate buffered saline (PBS) and incubated for an additional 24 h in serum-free DMEM. Ten milliliter of serum-free media was subsequently harvested, centrifuged at 2,000 × g to remove debris, and stored at –80°C. Prior to 2D-E, the samples were concentrated 100-fold using Vivaspin 10,000 molecular weight cut-off concentrators (Sartorius) and desalted with 2-D Clean-Up Kits (GE Healthcare Bio-Sciences, Uppsala, Sweden).

### Two-dimensional electrophoresis (2D-E)

2D-E was performed as previously described [11]. Briefly, proteins were rehydrated in 250 μl of rehydration buffer (8 M urea, 2 M thiourea, 4% CHAPS, 0.5% pharmalyte, 20 mM dithiothreitol) overnight in 13 cm precast immobilized dry strips at pH 4–7 (GE Healthcare Bio-Sciences). The strips were then subjected to isoelectric focusing (IEF) using the Ettan IPGphor 3 IEF System (GE Healthcare Bio-Sciences). They were subsequently equilibrated and subjected to second dimensional separation at 16°C using sodium dodecyl sulfate–polyacrylamide gel electrophoresis (SDS–PAGE) with 8–18% gradient gels. All samples were analyzed in technically triplicate.

### Silver staining

The 2D-E gels were silver stained as previously described by Heukeshoven and Dernick [12]. For mass spectrometric analysis, gels were silver stained as described by Shevchenko et al. [13].

### Concanavalin A chromatography

Ten milliliters of harvested medium was mixed with 2 ml of ConA Sepharose (GE Healthcare Bio-Sciences) and rotated overnight at 4°C. The mixture was then loaded into a column (0.8 cm × 4 cm) (Bio-Rad Laboratories, Hercules, CA, USA) and equilibrated with equilibration buffer (20 mM Tris-HCl, 0.5 M NaCl, pH 7.4). The column was washed with 50 ml of equilibration buffer to remove non-glycosylated and *O*-glycosylated proteins, and bound *N*-glycoproteins were eluted with 0.3 M methyl-α-D-glucopyranoside. Absorbance at 280 nm was measured throughout the chromatographic process. Eluted fractions were concentrated 100 times using Vivaspin concentrators (10,000 molecular weight cut-off; Sartorius) and further desalted with 2-D Clean-Up Kits (GE Healthcare Bio-Sciences) and then subjected to 2-DE analysis.

### Image analysis

Images of silver-stained 2D-E gels were captured using a LabScan scanner (version 5; Amersham, Germany), and PD-Quest™ 2D gel analysis software (version 8.0.1, Bio-Rad) was used to detect, match, and quantify protein spots. Normalization was achieved by dividing the raw quantity of each spot in the gel by the total intensity of pixels in the image in order to compensate for non-expression related variations in protein spot intensities. The variance ratio test (F) was used to analyse differences among the sample groups. A *p*-value less than 0.05 was considered statistically significant.

### Mass spectrometric analysis and protein identification

Highly resolved spots of interest were excised and subjected to in-gel tryptic digestion using ProteoExtract™ All-in-One Trypsin Digestion Kit (Calbiochem, Darmstadt, Germany). The trypsin-digested peptides were desalted and concentrated using C18 Zip Tip Pipette Tips (Merck Millipore, Billerica, MA, US). Mass spectrometric analysis was performed at the Proteomic Centre, Department of Biological Sciences, National University of Singapore. The digested peptides were mixed with 1 μl of CHCA matrix solution (5 mg/ml α-cyano-4-hydroxycinnamic acid in 0.1% trifluoroacetic acid and 50% acetonitrile in ultrapure water) and spotted onto a matrix-assisted laser desorption/ionization (MALDI) target plate. Mass spectra were obtained using an ABI 4800 Proteomics Analyzer MALDI-TOF/TOF Mass Spectrometer (Applied Biosystems, Framingham, MA, USA). The five most intense ions obtained from mass spectrometry (MS) were subsequently subjected to MS/MS analysis using air with collision energy of 2 kV and a collision gas pressure of ~1×10^-6^ Torr. Stop conditions specified 2,000–3,000 shots, depending on the quality of the spectra. For protein identification, mass spectra were compared with the National Center for Biotechnology Information non-redundant (NCBInr) protein database for *Homo sapiens* using the MASCOT search engine (version 2.1; Matrix Science, London, UK). Searches were performed with fixed modification on carbamidomethylation of cysteines and variable modification of methionine oxidation. The following parameters were used in the MASCOT peptide mass fingerprint search: (i) enzyme: trypsin; (ii) one missed cleavage allowed; (iii) mass value: monoisotopic; (iv) peptide mass tolerance: ±0.1 Da; and (v) peptide charge state: 1+. The same parameters were used in the MASCOT ion search, except peptide mass tolerance and fragment mass tolerance were set at 100 ppm and 0.2 Da, respectively. Search scores >50 indicated identity or extensive homology (p < 0.05).

## Results

### Comparison of the MCF-7 and HMEpC growth media profiles

**Figure 1:**
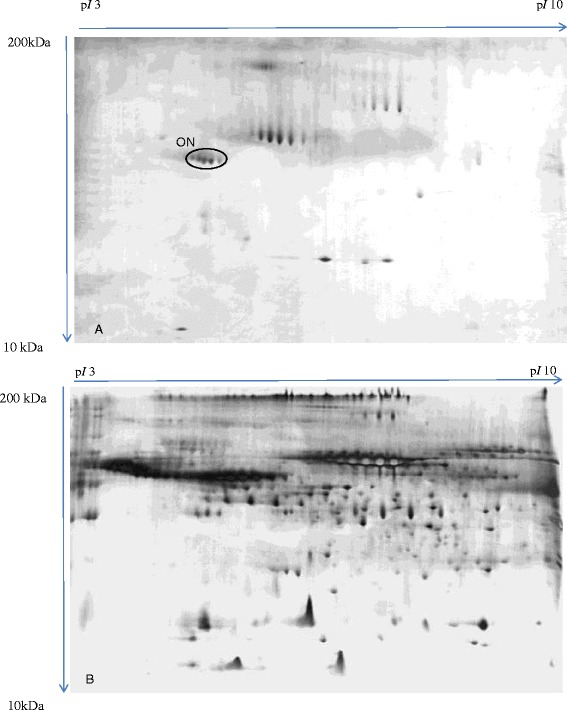
**Typical 2-DE growth medium profiles.** Panel **A** and panel **B** refer to typical 2-DE growth medium profiles of HMEpC and MCF-7 cell line, respectively. Acidic side of the gel is to the left and relative molecular mass declines from the top. ON was detected in the growth media of HMEpC cell line, but not in the growth media of MCF-7 cells.

Separation of MCF-7 media by 2D-E resulted in a high-resolution protein profile, which was comprised of various secreted factors (Figure 1B). However, we observed a considerably low proteins profile when HMEpC media was analyzed by 2D-E and silver staining. A protein cluster was solely detected in HMEpC media but was absent in MCF-7 media. It was then identified as osteonectin (Figure 1A and Table 1) when subjected to MS/MS analysis.

**Table 1:** **Mass spectrometric identification of protein spots from MCF-7 and HMEpC media using MASCOT search engine and the NCBI database**

**Spot name**	**Protein name**	**MASCOT accession number**	**Theoretical p** ***I***	**Theoretical mass (Da)**	**No. of peaks matched**	**Search score**
HP	Haptoglobin	gi|223976	6.23	42,344	2	70
ON	Osteonectin	gi|338325	4.70	35,260	1	81

### Detection of *N*-glycoproteins using ConA chromatography

**Figure 2:**
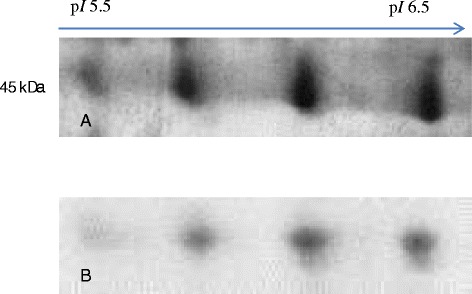
**Haptoglobin glycoforms.** Panel **A** and panel **B** demonstrates the cropped image of β chain of haptoglobin glycoforms obtained from 2-DE total growth medium profile and *N*-glycoproteins profiles of MCF-7, respectively. It is a 45 kDa homotetramer with p*I* ranged from 5.5 to 6.5.

We next investigated changes in oligosaccharide moieties on secreted proteins using a combined methodology, which coupled Con A chromatography for enrichment of *N*-glycoproteins based on alpha-mannose residues followed by 2-DE analysis. Using these techniques, we analyzed media harvested from MCF-7 and HMEpC cell lines and observed differential *N*-glycoprotein profiles. Image analysis of ConA chromatography of MCF-7 media demonstrated that the volumes of almost all matched spots were comparable except haptoglobin glycoforms, which was detected to be in low level compared to the total growth media profile (Figure 2 and Table 1). The volumes of other matched spots were comparable in both profiles. On the other hand, osteonectin was not observed in the *N*-glycosylation profile of HMEpC media although it is known to contain *N*-glycan (not shown) [14].

## Discussion

Breast cancer cells acquire aberrant protein expression, which can subsequently lead to alterations in post-translational modifications. Glycosylation is a common modification made to secreted proteins, which are involved in a variety of physiological processes, such as cancer cell signaling, matrix remodeling, and metastasis [15]. Therefore, changes in glycosylation or glycan composition occurring during tumorigenesis can ultimately contribute to cancer progression. In this study, we have analyzed the secretory profile of the MCF-7 breast cancer cell line in order to identify aberrantly secreted and/or glycosylated proteins.

Osteonectin (also known as secreted protein acidic and rich in cysteine [SPARC]) is a secreted, phosphorylated, and calcium-binding glycoprotein that was first isolated from bone [16]. It plays a role in regulating cell adhesion, proliferation, migration, and tissue remodeling [17]. Osteonectin overexpression was reported to be associated with malignancy in various tumors, including breast, brain, and prostate carcinoma [18]. However, there have also been reports of decreased osteonectin expression being associated with tumorigenesis in human ovarian cancer [19] and poor prognosis in breast cancer patients [20]. Thus, these studies have collectively suggested a context dependent role for osteonectin during tumorigenesis. We believe that these disparate findings might relate to osteonectin isoforms, which could be differentially expressed in various disease states. Our findings indicated that osteonectin was secreted from HMEpC, but not by MCF-7 cells. Osteonectin was not detected in *N-*glycoprotein profiles of HMEpC, although previous study has reported that it is a *N*-glycoprotein [14]. Future studies will be needed to determine the functional role and the *N*-glycosylation structure of osteonectin in breast cancer .

Haptoglobin is a glycoprotein that is mainly produced by hepatocytes. It binds to free hemoglobin to form stable haptoglobin–hemoglobin complexes that help to maintain iron ions in the blood. It also functions as an acute-phase reactant, and a tumor variant of haptoglobin exhibits immunosuppressor activity [21,22]. Structurally, haptoglobin is a 90 kDa tetramer composed of two non-identical α- and β-subunits linked by disulphide bonds [23]. In this study, we only analyzed the β-subunits of haptoglobin, which are glycosylated. Indeed, it was surprising to observe haptoglobin secretion by MCF-7 cells since it is a hepatocyte-secreted factor. Nevertheless, recent studies have observed haptoglobin expression associated with ovarian and breast tumors [21,22,24], as well as ovarian and embryonic lung cancer cell lines [25]. Moreover, previous reports by Kuhajda et al. [24] and Oh et al. [22] have indicated that the tumor variant of haptoglobin displays aberrant structural changes that may contribute to tumorigenesis. Since haptoglobin is a secreted protein, its level should be equal to its glycosylated forms [26]. However, the present findings demonstrated that β-haptoglobin glycoforms secreted by MCF-7 cells lacked *N*-glycosylation. This change in haptoglobin *N*-glycosylation could affect extracellular matrix interactions and/or contribute to immunosuppression of lymphocytes for promoting cancer. Therefore, we propose that the tumor variant of haptoglobin undergoes both structural and post-translational changes that are advantageous to cancer cells during tumorigenesis.

## Conclusion

Our combined secretomic and glycoproteomic study of the MCF-7 breast cancer cell line has yielded promising results. Comparative analyses have indicated that differentially glycosylated forms of haptoglobin were secreted by MCF-7 cells. This differentially secreted factor may play a role in tumorigenesis and could represent useful biomarkers for future breast cancer research and detection.
